# Toxicity of ivermectin on multiple insecticide-resistant populations of *Anopheles gambiae *sensu lato, *Aedes aegypti*, and *Culex* mosquitoes

**DOI:** 10.1186/s13071-025-07227-7

**Published:** 2026-01-21

**Authors:** Dorothy Obuobi, Godwin Kwame Amlalo, Andreas Wieser, Guenter Froeschl, Andreas Adutwum Kudom

**Affiliations:** 1 CIH LMU Center for International Health, University Hospital, LMU Munich, Munich, Germany; 2 Department of Conservation Biology and Entomology, University of Cape Coast, Cape Coast, Ghana; 3 Noguchi Memorial Institute for Medical Research, University of Ghana, Legon, Accra, Ghana; 4 Institute of Infectious Diseases and Tropical Medicine, Medical Center of the University of Munich, Munich, Germany; 5 German Center for Infection Research (DZIF), Partner Site Munich, Munich, Germany; 6 Max von Pettenkofer Institute of Hygiene and Medical Microbiology, Faculty of Medicine, LMU Munich, Munich, Germany; 7 Fraunhofer Institute for Translational Medicine and Pharmacology ITMP, Immunology, Infection and Pandemic Research, Munich, Germany

**Keywords:** Ivermectin, *Anopheles gambiae* sensu lato, *Aedes aegypti*, *Culex* species, Insecticide resistance, Metabolic resistance, Target-site mutation, Ghana

## Abstract

**Background:**

Ivermectin is an emerging vector control; however, its toxicity against insecticide-resistant mosquito populations with multiple resistance mechanisms remains unclear. This study investigated the toxic effects of ivermectin on three multiple insecticide-resistant mosquito populations from Ghana.

**Methods:**

Susceptibility to different insecticides, target-site mutations associated with insecticide resistance, and metabolic resistance mechanisms were determined among field mosquito populations of *Anopheles gambiae *sensu lato, *Aedes aegypti*, *Culex* species, and susceptible *Anopheles gambiae *sensu stricto* Kisumu* (laboratory strain). Dose–response bioassays were performed by feeding the mosquito populations with different concentrations of ivermectin dissolved in a 10% sugar solution. Mortality was recorded post-feeding every 12 h for 48 h.

**Results:**

The field mosquito populations were resistant to most of the insecticides tested, particularly the pyrethroids. Different *kdr* mutations and metabolic resistance mechanisms were detected in the field populations. The susceptible *An. gambiae* s.s.* Kisumu* strain had a significantly higher hazard of death compared with the insecticide-resistant *An. gambiae* s.l. (exhibiting *kdr*, *Ace-1* mutations and metabolic resistance mechanisms), across all the ivermectin concentrations (*P* ≤ 0.001). Furthermore, the lethal doses of ivermectin that killed 95% wild *An. gambiae* s.l. and *Culex* spp. (permethrin, deltamethrin, and dichloro-diphenyl-trichloroethane [DDT] resistant) were comparable, but lower than the dosage that killed 95% wild *Ae. aegypti* with *F1534C*, *V410L*, and *V1016I* mutations and metabolic resistance mechanisms.

**Conclusions:**

The study showed that the multiple insecticide-resistant *An. gambiae* s.l. population was more tolerant to ivermectin compared with the susceptible strain, but more susceptible to the drug compared with *Ae. aegypti*. These findings suggest heterogeneity in ivermectin responses across the mosquito species and resistant phenotypes, and therefore, further studies are needed to identify the mechanisms underlying these differences and to assess their relevance under broader epidemiological and ecological contexts.

**Graphical Abstract:**

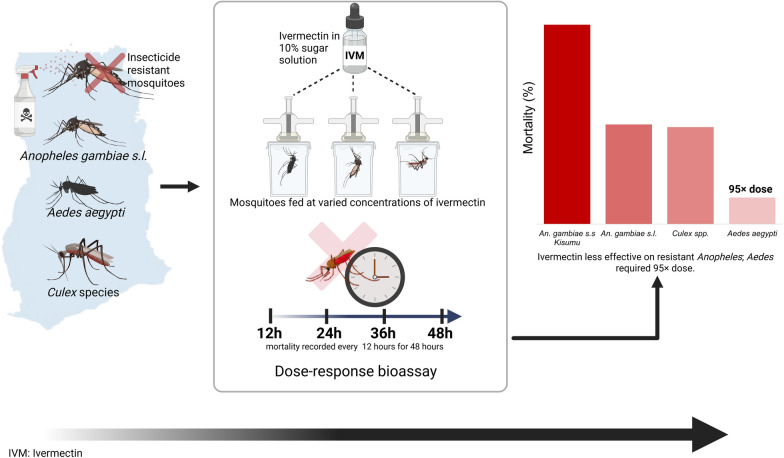

**Supplementary Information:**

The online version contains supplementary material available at 10.1186/s13071-025-07227-7.

## Background

The control of mosquito-borne diseases largely relies on the use of chemical insecticides to reduce mosquito populations and hence disease transmission [1]. The development of insecticide resistance in mosquito populations is threatening the effectiveness of major vector control measures, which are based on insecticides [2, 3].

Insecticide resistance in mosquito vectors is complicated and multidimensional, involving increased metabolic detoxification and decreased target-site sensitivity [4]. In the former, the overexpression of detoxifying enzymes breaks down insecticides before reaching their site of action. The three main enzyme families responsible for metabolic resistance are cytochrome P450s, glutathione *S*-transferases, and esterases [5]. Mutations such as knockdown resistance (*kdr*) and acetylcholinesterase (*Ace-1*), reduce insecticide efficacy, conferring resistance to pyrethroids, dichloro-diphenyl-trichloroethane (DDT), carbamates, and organophosphates [6, 7]. These mutations have been detected across many African countries, including Ghana [3, 8–10]. There is therefore an urgent need to develop and evaluate novel interventions to complement the existing tools to control insecticide-resistant mosquito vectors.

Ivermectin, which has been traditionally used to treat parasitic infections in humans and animals, has emerged as a promising vector control tool [11]. For over 20 years, it has been used in mass drug administration campaigns to control *Lymphatic filariasis* and onchocerciasis [12, 13]. Recent studies show that ivermectin can suppress mosquito populations, prevent oviposition, and reduce parasite and viral loads in mosquitoes [14–17]. This offers a new option for vector control. Unlike conventional insecticides, the effect of ivermectin is observed when ingested orally by mosquitoes as they feed on treated hosts, plants, and attractive toxic sugar baits (ATSB) placed in bait stations, reducing their lifespan and disease transmission potential [11, 18, 19].

Ivermectin exerts its toxic effects on invertebrates such as insects by preferentially binding to glutamate-gated chloride channels in invertebrate muscles and nerve cells, resulting in paralysis and death [20, 21]. The channel is not present in humans, and as such, ivermectin generally has a low-risk profile for humans while being toxic to mosquitoes and other arthropods [22]. Additionally, ivermectin has a different mode of action from conventional insecticides that are currently used [23]. Ivermectin has proven effective against *An. arabiensis*, *Ae. aegypti*, and *Culex* spp. [18, 24, 25].

However, the susceptibility of mosquitoes to ivermectin has been shown to be species- and strain-dependent. For instance, in Tanzania, ivermectin concentrations of 0.01% and 0.03% reduced *An. arabiensis* and *Ae. aegypti* populations by 95% and 90%, respectively, within 48 h [18, 24]. Also, when ingested, the median lethal concentration (LC)_50_ of ivermectin for *An. stephensi* was 0.0000007%, while that of *An. albimanus* was 0.0001468% [26]. This differential susceptibility calls for the need to characterize the major mosquito vectors in different geographic areas prior to using ivermectin for vector control. Additionally, a few studies have shown mosquito populations resistant to a single or double insecticides are less tolerant to ivermectin compared with the susceptible strain [15, 27]. However, the association between insecticide resistance mechanisms, including *kdr* and *Ace-1* mutations or elevated detoxification enzymes, and ivermectin toxicity remains poorly characterized. Understanding the impact of the current target-site mutations and metabolic resistance in mosquito vectors on the toxicity of ivermectin is crucial for any future use of ivermectin in vector control, particularly in regions where insecticide resistance is widespread.

To date, studies have yet to assess the toxicity of ivermectin across multiple mosquito species with well-characterized insecticide resistance profiles. This study was therefore conducted to evaluate how species-specific physiology and insecticide resistance mechanisms influence the toxicity of ivermectin by assessing its impact on *An. gambiae* s.l., *Ae. aegypti* and *Culex* spp. from Ghana with known multiple resistance mechanisms.

## Methods

### Mosquito sampling sites

**Fig. 1:**
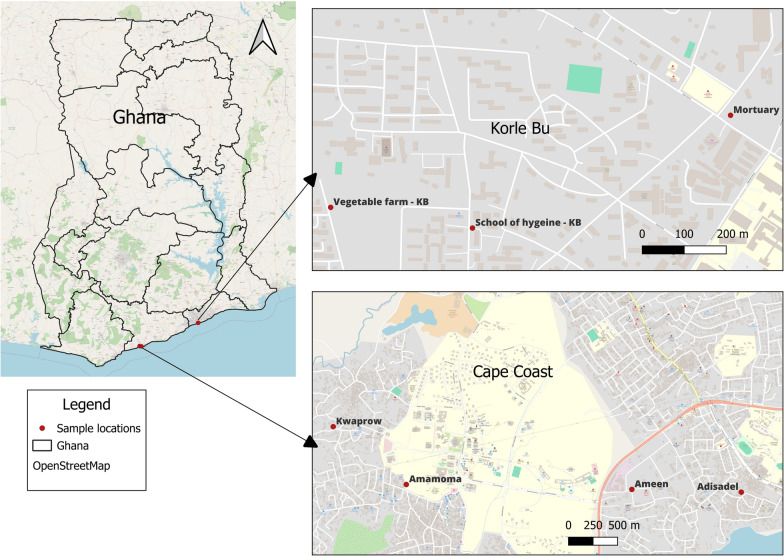
Map showing *An. gambiae* s.l., *Ae. aegypti*, and *Culex* spp. collection sites for the study. Source: http://openstreetmap.org/

The study was conducted in Cape Coast and Korle-Bu, two urban areas in the coastal savannah ecological zone of southern Ghana (Fig. 1). The selection of the study sites where the field mosquito populations were collected was based on our assumptions on the insecticide resistance status of the mosquitoes from previous studies. *An. gambiae* s.l. was collected in Cape Coast [28, 29], while *Ae. aegypti* and *Culex* spp. were collected in Accra [30, 31]. Cape Coast, the capital of the Central Region, covers over 120 km^2^ with a population of about 190,000 [32]. The peak rainfall season is between May and July with annual rainfall between 750 and 1000 mm. The average temperature ranges between 24 °C and 32 °C with mean monthly relative humidity ranging from 85% to 99%. Cape Coast has a hilly topography with the highest point of approximately 60 m above sea level with undulating valleys between the hills [33].

Fishing is a major occupation of the inhabitants, coupled with vegetable cultivation both commercial and backyard/home gardening as well as small-scale livestock rearing. Presently about 3500 hectares of land are under cultivation, and the commonly cultivated crops are lettuce, cabbage, eggplant, peppers, okra, and tomatoes [33]. Irrigation of these crops especially in the dry season from ponds, shallow wells, and stored rainwater provides breeding grounds for *An. gambiae* s.l. Also, human modifications, such as road construction, create rain-dependent, relatively clean pools and puddles in construction sites, serving as breeding sites for *An. gambiae* s.l. Additionally, the containers, buckets, and small household plots used for home gardening may provide breeding sites for *Ae. aegypti*, especially when the containers are left uncovered. Furthermore, in the livestock rearing, the watering of the animals, and their waste disposal may pollute nearby water bodies, making them suitable for *Culex* spp.

Korle-Bu, is located in Accra, the capital town of Ghana with an estimated population of 5 million [32]. Accra is a coastal town with an annual rainfall ranging from 730 to 1200 mm with the major rainy season from March to July and a minor season from September to November. The city has an average temperature between 24 °C and 29 °C and humidity of 77–85%. Korle-Bu is home to the largest hospital in Ghana and experiences higher temperatures due to limited greenery and has many vegetable farms situated in low-lying areas and along drains that collect stagnant or polluted water. The area is characterized by sandy-loamy soils and highly organic polluted drains from farm runoff and domestic wastes. Common crops cultivated include tomatoes, lettuce, cabbage, peppers, and carrots. The polluted drains and water-storage pits on the farms serve as breeding sites for *Culex* spp. The Korle-Bu locality is host to several vulcanizing shops and abandoned tires. Water in these tires serves as breeding sites for *Ae. aegypti* mosquitoes. The agricultural activities coupled with poor drainage and waste accumulation creates diverse larval habitats that support the breeding of multiple mosquito genera in the locality.

### Mosquito sampling

*An. gambiae* s.l., *Ae. aegypti*, and *Culex* spp. larvae were sampled from the study sites between December 2022 to November 2023. The long-handle ladle method was used to collect both *An. gambiae s.l.* and *Culex* spp. *Anopheles gambiae* s.l. larvae were collected from rain-dependent pools and puddles located in potholes and construction sites within Cape Coast, while *Culex* larvae were sampled from heavily polluted drains and water-storage pits in and around vegetable farms at Korle-Bu. *Aedes aegypti* larvae, however, were collected using a Pasteur pipette and a short-handled ladle from abandoned car tires around the mortuary and school of hygiene at Korle-Bu. All larvae were reared to adults in the insectary at the Department of Conservation Biology and Entomology following standard rearing protocol of the World Health Organization (WHO) [34]. Emerged adults were used for ivermectin susceptibility tests, WHO susceptibility tests, and biochemical assays.

### WHO susceptibility tube bioassays

WHO susceptibility bioassays were conducted using insecticide-treated papers (deltamethrin, permethrin, DDT, bendiocarb, and malathion) with different discriminating doses from four insecticide classes, purchased from a WHO-accredited institution (Universiti Sains Malaysia) (Table S1). Between 80 and 100 mosquitoes were evaluated per treatment. Batches of 20 or 25 non-blood-fed adult female mosquitoes (3–5 days old) of each species: *An. gambiae* s.l., *Ae. aegypti*, and *Culex* spp. were exposed to these papers in four replicate tubes for 1 h. After exposure, mosquitoes were transferred to holding tubes, and knockdown and mortality were recorded at 1 h and 24 h, respectively. However, the *Culex* spp. were exposed to permethrin and DDT for 3 h and 4 h, respectively. In addition, piperonyl butoxide (PBO) synergist bioassays were performed by pre-exposing mosquitoes to 4% PBO-treated papers before exposure to permethrin and deltamethrin. Additionally, four replicates of 20 mosquitoes each were exposed to PBO-only and oil-treated papers as controls. For molecular analysis, dead and surviving mosquitoes were stored in 1.5 mL microcentrifuge tubes at −20 °C. All tests followed the WHO’s standard protocols [35].

### Molecular analysis

Genomic DNA was extracted from individual dead and survived *An. gambiae* s.l. and *Ae. aegypti* specimens from the WHO susceptibility tests and ivermectin bioassays using the 2% cetyltrimethylammonium bromide (CTAB) protocol [36]. Species identification of *An. gambiae* s.l. and *Ae. aegypti* was confirmed using (polymerase chain reaction [PCR]) protocols by Scott et al. [37] and Higa et al. [38], respectively. Polymerase chain reaction products were visualized on a 2.0% agarose gel under ultraviolet light. Additionally, real-time PCR was performed to identify *An. gambiae* sensu stricto and *An. coluzzii* using the method described by Chabi et al. [39].

*An. gambiae* s.l. samples were screened for knockdown-resistance (*kdr*) *L1014F* and insensitive acetylcholinesterase (*Ace-1*) *G119S* mutations using real-time TaqMan assays described by Bass et al. [40, 41]. Each assay included a 20 μL reaction mix with DyNAmo single nucleotide polymorphism (SNP) master mix (2×; 10 μL), TaqMan probes (0.4 μL each), DNase-free water (4.5 μL), DNA (2 μL), and specific primers. For *kdr* *L1014F* and *ace-1* *G119S*, the mix contained 1.35 μL each of 10 μM forward primer (CATTTTTCTTGGCCACTGTAGTGAT) and the reverse primer (CGATCTTGGTCCATGTTAATTTGCA). The mix included 1.35 μL each of 10 μM forward primer (GGCCGTCATGCTGTGGAT) and reverse primer (GCGGTGCCGGAGTAGA). The PCR conditions were 10 min at 95 °C, followed by 40 cycles of 95 °C for 10 s, 65 °C for 40 s, and 72 °C for 30 s. Fluorescein amidite (FAM; 6-carboxyfluorescein) fluorescence showed the homozygous mutant allele (homozygous resistant genotype [RR]), fluorescence of the proprietary fluorescent dye VIC indicated the homozygous wild-type allele (homozygous susceptible genotype [SS]), and both signals indicated heterozygosity (heterozygous genotype [RS]). *Aedes aegypti* specimens were also genotyped for the *kdr* mutations, *F1534C* and *V1016I*, following the protocol of Estep et al. [42]. Additionally, a modified protocol of Saavedra-Rodriguez et al. [43] was used to detect the presence of the *kdr* mutation *V410L*. A melting curve produced at 82 °C and 78 °C indicated the mutant gene (resistant) and wild-type (susceptible), respectively, for *F1534C* gene. The mutant and wild-type genes, respectively, produced melting curve peaks at 76 °C and 83 °C for *V1016I* and peaks at 83 °C and 86 °C for *Vl410*.

### Metabolic detoxifying enzyme assays

A microplate assay was performed on 3–5-day-old field colonies of *An. gambiae* s.l. and *Ae. aegypti* to measure the activity of mixed function oxidase (MFO), esterases (α and β), glutathione-*S*-transferases (GST), insensitive acetylcholinesterase (AChE), and total proteins, following a modified Introduction to Microplate Enzyme Activity Assays protocol of the Malaria Research and Reference Reagent Resource Center (MR4) [34] and Leong et al. [44] protocol. Each enzyme was tested on 200 mosquitoes per species. The *Aedes aegypti* susceptible reference strain (a Cape Coast laboratory-reared susceptible strain) and susceptible *An. gambiae* s.s.* Kisumu* strain were used as controls for *Ae. aegypti* and *An. gambiae* s.l. populations, respectively. The *Ae. aegypti* Cape Coast susceptible strain was susceptible to pyrethroids (deltamethrin and permethrin), carbamate (bendiocarb), and organophosphate (fenitrothion). The resistance status against the organochlorine DDT was not specified.

Mosquitoes were homogenized in 1500 μL of potassium phosphate buffer on ice and centrifuged at 14,000 rpm for 1 min at 4 °C. The supernatant was used for enzyme assays. Assays were performed in duplicate on a 96-well microplate, with 100 μL of supernatant per well. Substrates and chromogenic agents specific to each enzyme were added. Positive controls were included for MFO and elevated esterase assays. Absorbance was measured using a Spectramax 340PC spectrophotometer with Softmax Pro 5.0 software. Enzyme activities were quantified as follows: MFO at 620 nm with 3,3′,5,5′-tetramethyl-benzidine dihydrochloride, acetylcholinesterase at 414 nm with acetylthiocholine iodide, GST at 340 nm with 1-chloro-2,4′-dinitrobenzene, and β-esterase at 540 nm with β-naphthyl acetate. Enzyme concentrations were extrapolated from standard curves, while GST and insensitive acetylcholinesterase levels were estimated using the Beer–Lambert law. Activities were normalized by the total protein content of each mosquito to account for size differences.

### Bioassay with carmoisine dye and chromotrope FB dye

Food dye was used to visualize the uptake of the ivermectin by the mosquito. To obtain an appropriate concentration that was not toxic but could persist for a longer period in the mosquito, a bioassay was conducted with two food dyes: carmosine dye and chromotrope FB dye. Mosquitoes were fed with serial concentrations of the dyes prepared with a 10% sugar solution. The mosquitoes were starved for 12 h: *An. gambiae* s.l. and *Culex* mosquitoes from 8:00 a.m. to 8:00 p.m., and *Ae. aegypti* mosquitoes from 8:00 p.m. to 8:00 a.m. Each of the three mosquito species was tested against the two dyes. Active, non-blood-fed female mosquitoes were transferred into bioassay paper cups 1 h before testing. Five replicates of 30 mosquitoes were tested per concentration. An additional five replicate cups, each containing 30 mosquitoes, were set up as negative controls. All the cups were monitored for moribund or damaged mosquitoes and replaced before the mosquitoes were fed with dye.

A 1.5 g cotton pad soaked in 20 mL of the test dye solution was placed on each labeled netted paper cup. Mosquitoes were allowed to feed on the dye solution for 12 h, while control groups were given only 10% sugar solution. After the feeding period, the number of mosquitoes with visibly red, engorged abdomens was recorded. From each replicate, 10 dye-fed mosquitoes were transferred to labeled paper cups, given a 10% sugar solution, and monitored daily for mortality for 7 days.

### Preparation of ivermectin solution

An injectable formulation of Intermectin^®^ (1.0%) was purchased from a local veterinary supplier for evaluation. The stock solution was serially diluted in a 10% sugar solution to obtain different concentrations, which were used to determine the lethal doses (LDs)—lethal dose for 25% of the population (LD_25_), median lethal dose (LD_50_), and lethal dose for 95% of the population (LD_95_)—for each mosquito species. To identify ivermectin-fed mosquitoes, 100 mg of chromotrope FB food-grade dyes were added to 100 mL of ivermectin solution for *Culex* spp. and *An. gambiae* s.l. tests. Mosquito abdomens were visualized for dye uptake. Carmosine dye (300 mg) was used to identify ivermectin-fed *Ae. aegypti*. The choice of dye and the concentration were based on the results from the dye bioassay that was conducted prior to this bioassay. Control samples for each species were fed a 10.0% sugar solution with dye as a negative control. Additionally, susceptible *An. gambiae* s.s.* Kisumu* strain was also exposed to ivermectin to serve as controls.

The bioassays were conducted in disposable paper cups covered with netting fastened by rubber bands to assess ivermectin’s impact on mosquito survival. Female, blood-naive mosquitoes (3–5 days old) of *An. gambiae* s.l., *Ae. aegypti*, and *Culex* spp. were starved for 12 h before testing. Twenty mosquitoes of each species were acclimatized in testing cups 1 h before exposure. The cups were monitored before administering ivermectin sugar-containing dye solutions to the mosquitoes. Any knocked down or moribund (unable to stand) mosquitoes were replaced. Baits, consisting of a 1.5 mg cotton roll soaked with 20 mL of the respective ivermectin dose solution (ivermectin + 10.0% sugar solution + dye), were placed on the mesh net cover of each labeled cup containing 20 mosquitoes. The baits were left for 12 h, from 8 a.m. to 8 p.m. for *Ae. aegypti* mosquitoes and from 8 p.m. to 8 a.m. for *An. gambiae* s.l. and *Culex* spp., respectively. Afterward, ivermectin-fed mosquitoes, identified by dyed engorged abdomens, were transferred to new cups, and cumulative mortality was recorded at 12, 24, 36, and 48 h post-feeding. The experiment was replicated 12 times for each of the concentrations per species. Cups were maintained at a temperature of 27 ± 2 °C and 75 ± 10% humidity. A mosquito was considered dead if it could not fly or stand and showed no signs of life. For each concentration, 12 replicates of 20 mosquitoes each were tested, except the 0.0016% for which 17 replicates were evaluated for *Culex* spp.

### Statistical analysis

The obtained datasets underwent normality test using the Shapiro–Wilk test. The phenotypic resistance level of mosquitoes was determined using WHO criteria: mortality ≤ 90% indicated resistance, mortality > 90% and <97% suggested suspected resistance, and mortality ≥ 98% indicated susceptibility. The differences in survival between the two *An. gambiae* s.l. strains on each concentration of ivermectin, as well as among *An. gambiae* s.l., *Ae. aegypti*, and *Culex* spp. on the different concentrations of carmosine and chromotrope dyes, respectively, were assessed using the log-rank test with the *survdiff* function. Hazard ratios (HR) and 95% confidence intervals (CI) were estimated using Cox proportional hazards models (*coxph* function) to quantify the effect of ivermectin on the survival of the *An. gambiae* s.l. strains. Assumptions of proportional hazards were tested using Schoenfeld residuals (*cox.zph* function). Bioassay data were also analyzed using the R package (version 1.1.0) to estimate the median lethal dose (LD_50_), lethal dose for 90% of the population (LD_90_), and LD_95_, and their 95% confidence intervals (CI). Additionally, the Mann–Whitney test was used to compare the median activities of each enzyme per mg of protein between the test population and the reference susceptible strains using the Stata software. Finally, the Fisher’s exact tests were used to determine the association between the *kdr* mutations and ivermectin toxicity. All statistical analysis were performed in R v. 4.2.3 (R Core Team, 2023) and Stata IC I5. A significance threshold of *P* < 0.05 was used for all the statistical tests. All graphs except the enzyme activity graphs were visualized using ggplot2 package in R (version 3.5.1).

## Results

### Mosquito species and abundance

A total of 19,480 mosquitoes were collected from various sites. This comprised of 5780 (29.7%) that were *Anopheles* species, 6120 (31.4%) *Aedes* species, and 7580 (38.9%) *Culex* species. All the *Anopheles* collected were *An. gambiae* s.l. consisting of *An. coluzzii* (58.2%), *An. gambiae* s.s. (27.9%), and hybrids (11.4%), with 2.5% unidentified. The *Aedes* mosquitoes were mainly *Ae. Aegypti* (98.0%), and the other species identified included *Ae. albopictus* (0.5%) and *Ae. vittatus* (1.0%). The remaining individual could not be identified (0.5%). The majority of the *Culex* spp. (70.0%) were morphologically identified as *Culex quinquefasciatus*. The remaining individuals could not be successfully identified to species level.

### WHO susceptibility bioassays

**Fig. 2:**
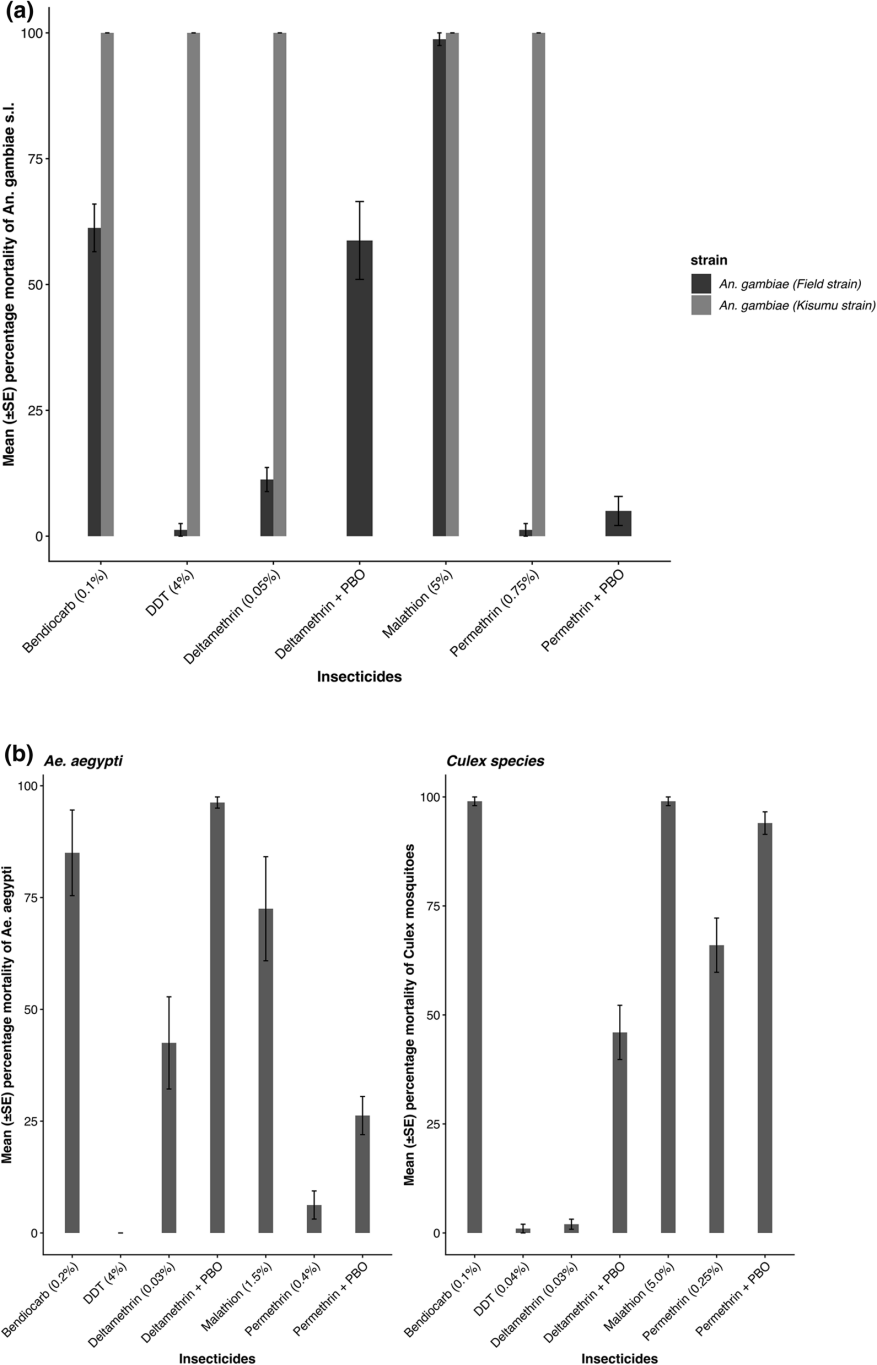
**a** Mean (± standard error [SE]) percentage mortality at 24 h of susceptible *An. gambiae* s.s. (*Kisumu* strain) and *An. gambiae* s.l. (field population), exposed to discriminating concentrations of insecticides in susceptibility tests. **b** Mean (±SE) percentage mortality at 24 h of field strains, *Aedes aegypti* and *Culex* spp., exposed to discriminating concentrations of insecticides in susceptibility tests

In total, 550 susceptible *An. gambiae* s.s *Kisumu* strain, 1120 each of field *An. gambiae* s.l. and *Ae. aegypti*, and 2000 *Culex* spp. were tested for susceptibility against the recommended insecticide dose. All the species showed varying resistance to pyrethroids, deltamethrin, and permethrin, with the highest resistance to permethrin (Fig. 2A, B). Pre-exposure to PBO improved mortality rates significantly (*χ*^2^ = 50.798, *df* = 10, *P* value = 1.903 × 10 ^−7^) but did not fully restore susceptibility. The field *An. gambiae* s.l. population was resistant to the pyrethroids (1.3–11.3%), DDT (0.0%), and bendiocarb (61.3%) but susceptible to malathion (98.8%). *Ae. aegypti* showed resistance to all insecticides (0.0–85.0% mortality), while *Culex* spp. were resistant to the pyrethroids (2.0–66.0%) and DDT (37.0%) but susceptible to bendiocarb (99.0%) and malathion (99.0%).

### Target-site mutations in *An. gambiae* s.l. and *Ae. aegypti* and association with pyrethroids

**Fig. 3:**
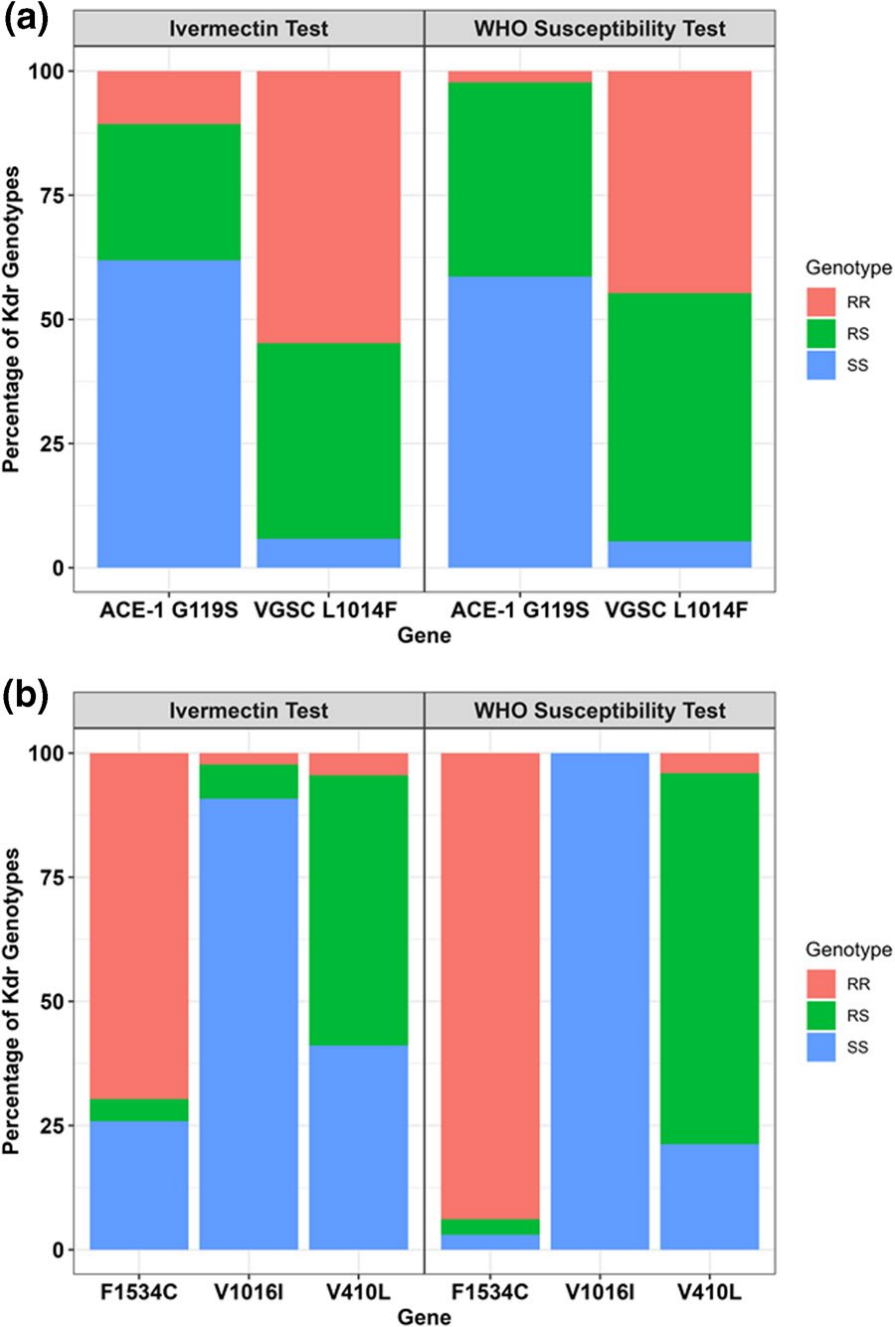
**a** Allele frequencies of *Kdr* mutations and *Ace-1* mutations in *Anopheles gambiae* s.l. mosquitoes from the ivermectin and WHO susceptibility tests. **b** Allele frequencies of *Kdr* mutations in *Aedes aegypti* mosquitoes from the ivermectin and WHO susceptibility tests

The assessment of two target-site mutations in the *An. gambiae* s.l. revealed a high frequency of *Vgsc-L1014F* *Kdr* mutation (71.8%, *N* = 179) and a lower frequency of *G119S* *Ace-1* mutation (23.2%, *N* = 170) (Fig. 3A, B). In the *An. gambiae* s.l. population, the resistant *kdr* genotypes (RS + RR) were present in 94.2% and 94.6% of the mosquitoes used for the ivermectin test and WHO susceptibility test, respectively. Conversely, the prevalence of *Ace-1* mutation was 38.1% and 41.9% in the ivermectin and WHO test samples, respectively (Fig. 3A). The *L1014F* *kdr* mutation was not significantly associated with the pyrethroids (Fisher’s exact test value = 3.294, *P* < 0.166). In the *Ae. aegypti* mosquitoes, out of the 189 genotyped samples, the mutant allele, *F1534C*, was the most dominant, as it was present in 84.2% of *Ae. aegypti* analyzed, followed by the *V410L* allele, which was present in 36.8% of the population, with the *V1016I* allele occurring in only 1.6% of the *Ae. aegypti* assayed (Fig. 3B). No association was observed between the pyrethroids and *F1534C* *kdr* mutation (Fisher’s exact test value = 2.394, *P* < 0.31 7) and *V410L* *kdr* mutations (Fisher’s exact test value = 1.373, *P* < 0.772).

### Metabolic detoxifying enzyme assays

**Fig. 4:**
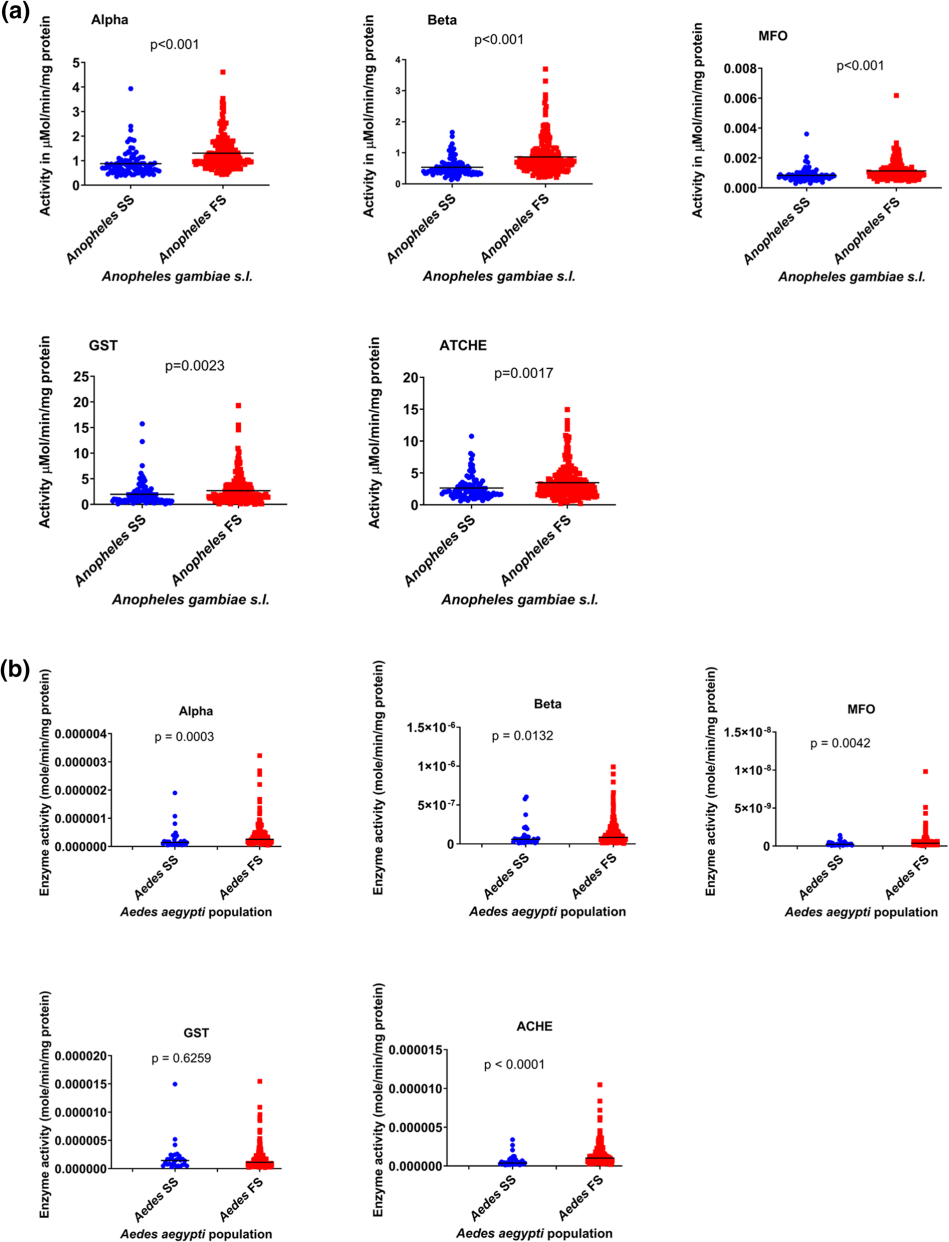
**a** Comparison of mean enzyme activities between susceptible *An. gambiae* s.s. (*Kisumu* strain) and *Anopheles gambiae* s.l. (field population) with the mean activity of the *Kisumu* susceptible strain used as the cut-off point. SS denotes susceptible strain and FS denotes field sample. **b** Comparison of mean enzyme activities between susceptible *Aedes aegypti* (Cape Coast strain) and *Aedes aegypti* (field population) with the mean activity of the susceptible strain used as the cut-off point. SS denotes susceptible strain and FS denotes field population

The *An. gambiae* s.l. and *Ae. aegypti* field populations were compared with susceptible *An. gambiae* s.s.* Kisumu* strain and *Ae. aegypti* Cape Coast susceptible strain, respectively. Overall, the metabolic enzyme assays showed significantly higher median activities of all five enzymes tested in the field *An. gambiae* s.l. population compared with susceptible *An. gambiae* s.s.* Kisumu* strain (*P* < 0.002, Fig. 4A). This suggests greater metabolic activity in the *An. gambiae* s.l. Similarly, the median activity of all the enzymes assayed, except for GST in the *Ae. aegypti* population was significantly elevated relative to the *Ae. aegypti* Cape Coast susceptible strain (*P* < 0.01, Fig. 4B), indicating high metabolic activity in the *Ae. aegypti* field population.

### Impact of the dyes on the survival of *Ae. aegypti*, *An. gambiae* s.l., and *Culex* spp.

Overall, 450 mosquitoes were evaluated for each of the species. There was no significant difference in the survival probability of *Ae. aegypti* exposed to the control and different concentrations of carmosine (100 mg, 200 mg, and 300 mg; *χ*^2^ = 2.3, *P* = 0.5, *df* = 3) and chromotrope (100 mg, 200 mg, and 300 mg; *χ*^2^ = 0.6, *P* = 0.9, *df* = 3), respectively; *Culex* spp. exposed to control and different concentrations of carmosine (*χ*^2^ = 3.4, *P* = 0.3, *df* = 3) and chromotrope (*χ*^2^ = 4.7, *P* = 0.2, *df* = 3), respectively; and *An. gambiae* s.l. exposed to the control and different concentrations of carmosine (*χ*^2^ = 2.3, *P* = 0.5, *df* = 3). The two dyes at the three concentrations used did not affect the survival of the mosquitoes compared with the control.

### Toxicity of ivermectin against *An. gambiae* s.l., *Ae. aegypti*, and *Culex* spp.

**Fig. 5:**
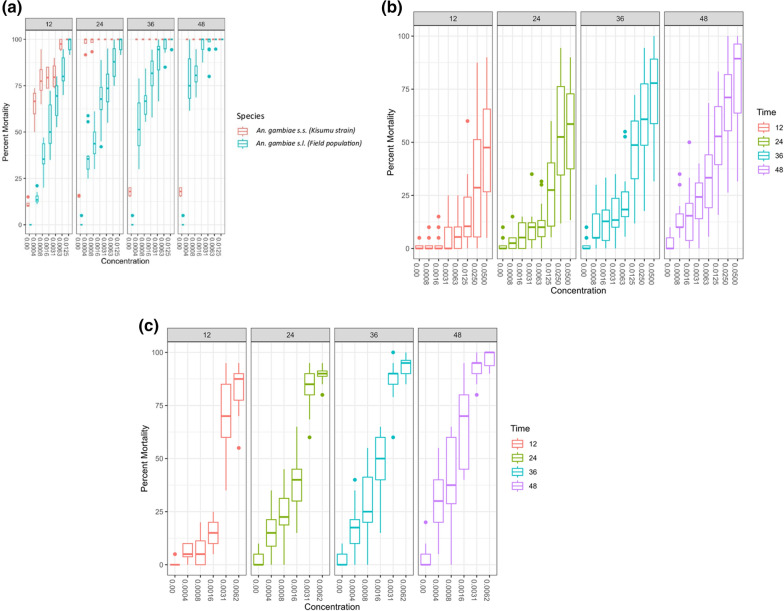
**a** Boxplots showing the percent mortality of susceptible *An. gambiae* s.s. (*Kisumu* strain) and insecticide-resistant *An. gambiae* s.l. (field population) at 12, 24, 36, and 48 h after exposure to different ivermectin concentrations in 10.0% sugar solution. **b** Boxplots showing the percent mortality of insecticide-resistant field population of *Aedes aegypti* at 12, 24, 36, and 48 h after exposure to different ivermectin concentrations in 10.0% sugar solution. **c** Boxplots showing the percent mortality of insecticide-resistant field population of *Culex* spp. at 12, 24, 36, and 48 h after exposure to different ivermectin concentrations in 10.0% sugar solution

In total, 1680, 1540, and 1920 mosquitoes of *An. gambiae* s.l., *Culex* spp, and *Ae. aegypti*, respectively, were tested. Ivermectin was toxic to all the mosquito species evaluated in this study. Dose-mortality responses between 12 and 48 h have been summarized in Fig. 5A–C. *An. gambiae* s.l. was more susceptible to the drug than *Culex* spp. or *Ae. aegypti*, while *Ae. aegypti* was more tolerant to the drug (Table 1). The lethal dose of ivermectin that killed 25% of *Ae. aegypti* was 32 times higher than that of *An. gambiae* s.l. and 9 times higher than that of *Culex* species. Notwithstanding, the lethal dose of the ivermectin that killed 95% of *An. gambiae* s.l. or *Culex* was the same, but 95 times lower than the dosage that killed 95% of *Ae. aegypti* (Table 1).

**Table 1:** Comparative toxicities of ivermectin to *An. gambiae* s.l., *Ae. aegypti*, and *Culex* spp. when ingested orally in 10.0% sugar solution

Mosquito species	LD_25_ (95% CI)	LD_50_ (95% CI)	LD_95_ (95% CI)
*An. gambiae s.l.*	0.0002 (0.0000–0.0009)	0.0005 (0.0004–0.0024)	0.0087 (0.0018–0.0296)
*Ae. aegypti*	0.0064 (0.0016–0.0188)	0.0263 (0.0081–0.0662)	0.8257 (0.4089–1.4400)
*Culex* spp.	0.0007 (0.0002–0.0023)	0.0015 (0.0004–0.0046)	0.0087 (0.0027–0.0231)

*LD* lethal dose, *CI* confidence interval

### Survival of different strains of *An. gambiae* s.l. following exposure to different concentrations of ivermectin

There was a significant difference in the survival probability of *An. gambiae* s.l. (field population) and susceptible *An. gambiae* s.s.* Kisumu* strain, (reference strain) among the different concentrations of ivermectin over time (*χ*^2^ = 1567, *P* ≤ 0.001, *df* = 11). The survival probability of susceptible *An. gambiae* s.s.* Kisumu* strain (reference strain) decreased rapidly over time across all the concentrations of ivermectin relative to *An. gambiae* s.l. (field population) (Fig. 5A). The Cox proportional hazards regression model was significant (likelihood test = 349.2, *P* ≤ 0.01). The susceptible *An. gambiae* s.s.* Kisumu* strain (reference strain) had a significantly higher hazard of death compared with *An. gambiae* s.l. (field population) across all the concentrations of the ivermectin (*B* = 1.21, standard error [SE] = 0.06, HR = 3.34, 95% CI [2.97, 3.76], *P* ≤ 0.001) (Fig. 5A).

### Association of *L1014Fkdr* mutation in *An. gambiae* s.l. with ivermectin

Genotyping of the *An. gambiae* s.l. species for the *L1014F* mutation revealed that the homozygous resistant genotype of *L1014F* mutation (LL) was dominant in both the dead and surviving mosquitoes across most ivermectin concentrations. At 0.00039% and 0.00078125% concentrations, the LL genotype remained dominant in the dead (60.0% and 77.8%) and surviving (75.0% and 62.5%) mosquitoes, respectively. At the 0.00015625%, the LL genotype was the most dominant in the dead (45.5%), whereas the heterozygous resistant genotype of *L1014F* mutation (LF) was common among the survivors (57.1%). Likewise, at the 0.003125%, the LF genotype dominated in both the dead (53.3%) and surviving groups (66.7%). Overall, the LL genotype predominated both dead (57.1%) and surviving groups with (55.6%) across all ivermectin concentrations. No significant association was observed between the *L1014F* genotypes and phenotypic resistance across all ivermectin concentrations (Fisher’s exact test = 1.147, *P* = 0.670).

### Association of *F1534C*, *V1016I*, and *V410Lkdr* mutations in *Ae. aegypti* with ivermectin

#### *F1534C* mutation

The homozygous resistant CC genotype was dominant in both the dead and surviving mosquitoes across all ivermectin concentrations, including 0.05% (dead: 50.0%; survivors: 100.0%). An exception was observed at 0.00078% concentration, where homozygous susceptible genotype of *L1014F* mutation (FF) was predominant in the dead mosquitoes (70.0%), whereas CC genotype dominated in the survivors (100.0%). Overall, the CC genotype dominated in both groups (dead: 95.0%; survivors: 61.4%). No significant association was observed between the genotypes of *F1534* and phenotypic resistance at individual concentrations (0.05%, *P* = 0.576), but a significant overall association was observed across all ivermectin concentrations (Fisher’s exact test = 11.013, *P* < 0.002).

#### *V410L* mutation

Analysis of the *V410L* mutation showed that the heterozygous resistant VL genotype was the most common among the dead mosquitoes, whereas the homozygous resistant VV genotype dominated the survivors at 0.05% ivermectin concentration (80.0% and 100.0%, respectively). This trend was observed across all concentrations except for 0.0125%, where the genotype was dominant among the dead mosquitoes and the LL genotype dominated the survivors. In total, VL genotype was predominant in the dead group and VV genotype among the surviving mosquitoes (70.0% and 90.0%, respectively) across all ivermectin concentrations. A significant association was observed between the genotypes of *V410L* and phenotypic resistance at 0.0125% (*P* < 0.045), 0.0250% (*P* < 0.015), and 0.0016% concentrations (*P* < 0.015). In contrast, no association was found at concentrations of 0.0500% (*P* < 0.091), 0.0063% (*P* < 0.455), 0.0031% (*P* < 0.105), and 0.0007% (*P* < 0.070). Nevertheless, generally, a significant association was observed between the genotypes of *V410L* and phenotypic resistance of ivermectin (Fisher’s exact test = 35.428, *P* < 0.000).

#### *V1016I* mutation

The homozygous resistant VV genotype was dominant in both the dead (90.0%) and surviving mosquitoes (100.0%), at 0.05 concentration of ivermectin. This pattern was observed at all individual concentrations. Overall, the VV genotype dominated in the dead (92.9%) and survivors (77.8%) across all ivermectin concentrations. There was no association observed between the genotypes of *V1016I *and phenotypic resistance at 0.05% ivermectin concentration. This pattern was consistent at all individual concentrations except at 0.0125% concentration (*P* < 0.045). Overall, a significant association was observed between *V1016I* genotypes and phenotypic resistance across all ivermectin concentrations evaluated (Fisher’s Exact Test = 6.480, *P* < 0.035).

## Discussion

The field mosquito populations used in this study were all resistant to pyrethroid insecticides and DDT, with varying degrees of resistance to carbamate and organophosphate insecticides. In addition, the mosquito populations had both target-site mutation and metabolic resistance mechanisms. The *An. gambiae* s.l. population had both *kdr* and *Ace1* mutations, while *Ae. aegypti* had three *kdr* mutations, and both mosquito populations had varying levels of enzyme activities. *Culex* mosquitoes were not screened for target-site mutation, nor was their level of enzyme activity determined. However, the results from the susceptibility test against permethrin and deltamethrin alone and plus PBO suggest the presence of both *kdr* and metabolic resistance mechanisms in the *Culex* populations. A previous study in the same study site detected *kdr* and *Ace-1* mutations as well as elevated enzyme activities in the *Culex* populations [45].

The ivermectin showed a strong toxicity to the multiple insecticide-resistant mosquito populations used in this study. A clear dose–response relationship was observed across all the species; higher concentrations and longer exposure periods led to increased mortality. However, tolerance to ivermectin differed among the different mosquito species as well as insecticide-susceptible and multiple insecticide-resistant strains. *Aedes aegypti* population was extremely tolerant to ivermectin compared with *An. gambiae* s.l. and *Culex* populations. *Ae. aegypti* higher tolerance to ivermectin relative to *An. gambiae* s.l. has also been shown in other studies [46, 47]. In Kobylinski et al. [46], where the resistance statuses of the mosquito populations were unknown, the lethal concentration, LC_50_ of ivermectin fed to *An. gambiae* s.s. was 0.00000224%, while *Ae. aegypti* was 0.00006013%. However, it has been shown that, due to physiological differences in the midgut epithelium among *Anopheles*, *Aedes*, and *Culex* species, glutamate-gated chloride (*GluCl*) is differentially expressed across these mosquito species [48]. These physiological differences may account for the differential tolerance to ivermectin among the three mosquito species since ivermectin resistance in other arthropods is associated with mutations involving *GluCls* [49].

Within the *An. gambiae* s.l. populations, the multiple insecticide-resistant population was more tolerant to the ivermectin compared with the susceptible population. The survival probability of susceptible *An. gambiae* s.s.* Kisumu* strain (reference strain) decreased rapidly over time across all the concentrations of the ivermectin relative to the multiple insecticide-resistant population (field population). A study by Kumar et al. [15] also found that the *An. culicifacies* and *An. stephensi*, resistant to DDT and malathion, were about two times more tolerant to ivermectin than the susceptible strains for each species. A similar result was reported by Deus et al. [27] in *Ae. aegypti*, where the three permethrin-resistant strains were significantly less susceptible to ivermectin than the susceptible reference strain after they were all fed with blood containing ivermectin.

Owing to the different target sites of the conventional insecticides and ivermectin, the tolerance of the multiple resistant strains to ivermectin suggest a possible role of metabolic resistance mechanisms. Different studies in *An. gambiae* s.l. mosquitoes have found that inhibiting the cytochrome P450 and *P*-glycoprotein resulted in increased mosquito mortality [50, 51], strengthening the evidence of the role of these transporters and enzymes in ivermectin detoxification. From this study, the field *An. gambiae* s.l. and *Ae. aegypti* strains had elevated activity for esterases and MFO metabolic enzymes, which may have contributed to the tolerance exhibited by the *Ae. aegypti* population to ivermectin. Similarly, previous studies have linked ivermectin resistance to mainly elevated activities of cytochrome P450 enzymes and ABC transporters in *An. gambiae* s.s. and *Rhipicephalus (Boophilus) microplus* ticks respectively [51, 52]. This could explain the differences in susceptibility between the resistant and the susceptible strains of *An. gambiae* s.l. from this study.

Genotyping of the target-site mutations revealed species-specific patterns. The *L1014F* *kdr* mutation was highly predominant in the *An. gambiae* s.l. population, with the homozygous resistant (LL) genotype as the most common in both dead and surviving mosquitoes across all ivermectin concentrations. However, no association was observed between the genotypes of *L1014F* and ivermectin resistance, suggesting that this mutation may not confer cross-resistance to ivermectin. However, the three *kdr* mutations detected in the *Ae. aegypti* population, showed overall significant associations with ivermectin resistance—*V1016I* (*P* = 0.035), *V410L* (*P* < 0.001), and *F1534C* (*P* = 0.002). These mutations may have contributed to the tolerance of the *Ae. aegypti* population to ivermectin and could contribute to partial cross-resistance between pyrethroids and ivermectin in multiple resistant *Ae. aegypti* mosquitoes. Notwithstanding, the study did not assess target-site mutations in the* GluCl* gene of the field mosquitoes. There are limited studies on target-site ivermectin resistance mechanisms in arthropods [53]. A study showed that resistant head lice had genetic mutations in the *GluCl* gene [54]. But the follow-up study was not consistent with the earlier study [55]. Also, Meyers et al. [48] suggested that resistance to ivermectin could arise through altered regulation of the *GluCl* splicing in *An. gambiae* s.s. These findings emphasize the need to investigate the role of target-site mutations in the *GluCl* gene among mosquitoes that exhibit more tolerance to ivermectin.

Furthermore, ivermectin has been explored as a potential complementary vector control tool, particularly in communities threatened by insecticide resistance. However, the differential toxicity among the different mosquito species, as well as in resistant and susceptible strains, may affect the drug’s effectiveness.

This study was unable to investigate the insecticide resistance mechanisms (resistant genotypes and metabolic enzymes) in the *Culex* spp. We were only able to investigate the phenotypic resistance, as we could not obtain positive DNA controls required for PCR assays for species identification and resistance mutation detection during the study period. Additionally, genotypic analysis of *Culex* spp. required extended protocol optimization. As this was our laboratory’s first attempt to investigate this species at the molecular level, initial PCR assays yielded no diagnostic bands, and the optimization process could not be completed within the study’s timeline.

## Conclusions

The study identified differences in ivermectin susceptibility among the mosquito populations evaluated. The multiple insecticide-resistant *An. gambiae* s.l. population was more tolerant to ivermectin compared to the susceptible strain, but more susceptible to the drug compared to the multiple insecticide-resistant *Ae. aegypti* population. These findings suggest heterogeneity in ivermectin responses across the mosquito species and resistant phenotypes. Further studies are required to identify the mechanisms underlying these differences and to assess their relevance under broader epidemiological and ecological contexts. These findings will be shared with relevant stakeholders involved in mosquito control, particularly in Ghana, for decision-making.

## Supplementary Information

Additional file 1.

## Data Availability

Data supporting the main conclusions of this study are included in the manuscript.

## Abbreviations

P450: Cytochrome P450 monooxygenases
*Kdr*: Knockdown resistance
*Ace-1*: Acetylcholinesterase-1 gene
DDT: Dichloro-diphenyl-trichloroethane
ATSB: Attractive toxic sugar bait
WHO: World Health Organization
PBO: Piperonyl butoxide
DNA: Deoxyribonucleic acid
CTAB: Cetyl trimethyl ammonium bromide
PCR: Polymerase chain reaction
SNP: Single nucleotide polymorphism
FAM: Fluorescein amidite
RR: Homozygous resistant genotype
SS: Homozygous susceptible genotype
RS: Heterozygous genotype
MFO: Mixed function oxidases
GST: Glutathione-*S*-transferases
AChE: Acetylcholinesterase enzyme
LD: Lethal dose
LC: Lethal concentration
LL: Homozygous resistant genotype of L1014F mutation
LF: Heterozygous resistant genotype of L1014F mutation
FF: Homozygous susceptible genotype of L1014F mutation
CC: Homozygous resistant genotype of F1534 mutation
FF: Homozygous susceptible genotype of F1534 mutation
VV: Homozygous resistant genotype of V410L mutation
LL: Homozygous susceptible genotype of V410L mutation
GluCl: Glutamate-gated chloride channel
ABC: ATP-binding cassette transporters
