# Supplementary material for: Toxicity of ivermectin on multiple insecticide-resistant populations of Anopheles gambiae sensu lato, Aedes aegypti, and Culex mosquitoes
Source: Parasit Vectors. 2026 Jan 21;19:83. doi: 10.1186/s13071-025-07227-7 (PMC12906033; doi:10.1186/s13071-025-07227-7)
Supplement: Supplementary file 1 — Additional file 1. [file 13071_2025_7227_MOESM1_ESM.docx]

**Table S1. Insecticide discriminating concentrations used for WHO susceptibility bioassays with *An. gambiae*, *Ae. aegypti, and Culex* species**

|  | | **Discriminating concentrations** | | |
| --- | --- | --- | --- | --- |
| **Insecticide class** | **Insecticide** | ***An. gambiae*** | ***Ae. aegypti*** | ***Culex* spp.** |
| Pyrethroids | Permethrin | 0.75% | 0.40% | 0.25% |
|  | Deltamethrin | 0.05% | 0.03% | 0.03% |
| Carbamate | Bendiocarb | 0.10% | 0.20% | 0.10% |
| Organochlorine | DDT | 4.00% | 4.00% | 0.04% |
| Organophosphate | Malathion | 5.00% | 1.50% | 5.00% |
